# Sarcopenic Characteristics of Active Older Adults: a Cross-Sectional Exploration

**DOI:** 10.1186/s40798-021-00323-9

**Published:** 2021-05-17

**Authors:** Zoya Huschtscha, Alexandra Parr, Judi Porter, Ricardo J. S. Costa

**Affiliations:** 1 Department of Nutrition Dietetics & Food, Monash University, Level 1, 264 Ferntree Gully Road, Notting Hill, Victoria 3168 Australia; 2 School of Exercise and Nutrition Sciences, Deakin University, Melbourne Burwood Campus, 221 Burwood Highway, Burwood, Victoria 3125 Australia

**Keywords:** Strength, Power, Cardiorespiratory, Protein, Exercise, Body composition

## Abstract

**Background:**

Ageing is associated with a decline in skeletal muscle mass and function (strength and power), known as sarcopenia. Inadequate dietary protein and inactivity have been shown to accelerate sarcopenia outcomes, occurring at different rates in males and females. Regardless, active older adults who often exceed the exercise guidelines still show signs of sarcopenia. This study aimed to explore the link between age, physical activity, protein intake, and biological sex with skeletal muscle mass, strength, power, and physical capacity/performance in active older adults. Fifty-four active older adults were recruited from this trial and grouped according to age (middle aged: 50–59 years, and older age: ≥ 60 years), exercise volume (low: ≥ 90–149 min/week, moderate: ≥ 150–299 min/week, and high: ≥ 300 min/week), protein intake (low: < 0.8 g/kg body mass (BM), moderate: ≥ 0.8–1.19g /kg BM, and high: ≥ 1.2 g/kg BM), and biological sex (males and females). Skeletal muscle and fat mass (dual X-ray absorptiometry), strength (1-repetition maximum using leg press, chest press, lateral pull down, and hand grip), power (counter movement jump), and general fitness (cardiorespiratory capacity and gait speed) were assessed. Data were grouped based on variables, and a general linear model (ANCOVA) or an independent *t* test was used to determine between group differences.

**Results:**

Fifty three of the total participants’ data were analysed. The middle-aged group had 18%, 11%, and 10% higher leg press, chest press, and lateral pull down, respectively, compared to the older-aged group (*p* < .05). There were no significant differences between different levels of training volume and any of the outcomes. Higher protein intakes were associated with significantly less body fat mass (*p* = .005) and a trend towards a higher leg press (*p* = .053) and higher relative power (W/kg) (*p* = .056) compared with the moderate and low protein intake groups. Significant differences based on biological sex were observed for all outcomes except for gait speed (*p* = .611) and cardiorespiratory fitness (*p* = .147).

**Conclusions:**

Contributions of age, physical activity, daily protein intake, and biological sex can explain the individual variation in outcomes related to changes in body composition, strength, power, and/or cardiorespiratory fitness in a cohort of active older adults.

The preprint version of this work is available on Research Square: https://www.researchsquare.com/article/rs-51873/v1.

**Trial Registration:**

This trial is registered in the ANZCTR.org.au, no. ACTRN12618001088235 (https://www.anzctr.org.au/Trial/Registration/TrialReview.aspx?id=375286).

**Supplementary Information:**

The online version contains supplementary material available at 10.1186/s40798-021-00323-9.

## Key Points

- There appears to be a significant difference in whole body strength outcomes in middle-aged older adults (50–59 years) compared with older adults (≥ 60 years) that are considered active.
- There were significant differences in outcomes of body composition, strength, and performance observed based on biological sex.
- Active older adults have a low risk of adverse outcomes caused by age-related sarcopenia.

## Background

The global population is ageing and older adults (i.e., middle-aged: ≥ 50–59 years; older: ≥ 60 years) are remaining active much later in life than in previous generations. This is evident in the growing participation rates of both recreational and competitive older adults in endurance events, such as marathons [1] and ultra-endurance marathons [2, 3]. Many studies report that regular physical activity is imperative to mitigate age-related declines in skeletal muscle mass (SMM), strength, power, and general physical performance—known as sarcopenia [4, 5]. For example, a systematic review and meta-analysis of 14 studies concluded that older adults engaging in regular physical activity reduced the odds of acquiring sarcopenia later in life (odds ratio [OR] = 0.45; 95% confidence interval [CI] 0.37–0.55) [6]. However, active older adults still show signs of declining physical function with increasing age despite often exceeding the physical activity guidelines and exercising significantly more than sedentary older adults (> 150 min/week of moderate intensity or 75 min/week of vigorous intensity—or combination) [7, 8]. For example, highly competitive Masters level athletes (≥ 35 years) show declines of decreased cardiorespiratory fitness (e.g., $\dot{V}{\mathrm{O}}_2$) and peak power compared with younger trained athletes (i.e., 18–27 years) in a variety of sporting disciplines [4, 9]. This decline in physical performance is most notable from the age of 50 years (middle-aged) until the age of 60–70 years (older) where it declines exponentially thereafter [10]. Despite this, the results in recreationally active and Masters athletes regarding SMM and function have been conflicting. Such differences are likely due to small sample sizes, few female athletes, limited outcome assessments (e.g., biochemistry markers), and not taking into consideration lifestyle factors (e.g., not assessing dietary intakes). The rates of decline in SMM and strength are often highly individual and been attributed to intrinsic (e.g., hormonal changes) and extrinsic (e.g., lifestyle choices and social influences) factors, in which physical activity habits, biological sex, and dietary habits play a key impacting role [11]. Despite this, active older adults, while currently underrepresented in sarcopenia research, may provide the ideal cohort to study as they do not display the extrinsic factor of sedentary behaviour often seen in sarcopenia research [12]. Therefore, understanding how age, habitual exercise, protein intakes, and biological sex may influence outcomes of body composition, strength, power, and physical performance (e.g., cardiorespiratory fitness and gait speed) in an active cohort may provide guidance for future intervention studies.

Although the causal mechanisms of sarcopenia remain elusive, it is likely multifactorial; these include decreases in anabolic hormones (e.g., testosterone), increased low-grade chronic inflammation, loss of neuromuscular function, mitochondrial dysfunction, infiltration of lipids within the skeletal muscle, and changes in muscle protein balance [12, 13]. Therefore, it is likely that the causes outlined, create a disruption of skeletal muscle protein turnover, resulting in an imbalance between muscle protein synthesis (MPS) and muscle protein breakdown (MPB), leading to loss of skeletal muscle mass and ultimately muscular function [14]. For example, a 12-year longitudinal study observed a 12.5% decline in the cross-sectional area (CSA) of the thigh muscle and a 21–35% loss of leg strength of healthy older adults (initial age (mean ± SD): 65.2 ± 4.2 years) [15]. However, studies in recreationally active and Masters athletes in regards to SMM have been conflicting. One cross-sectional study found that strength- or power-trained Masters athletes (i.e., 68 ± 0.8 years) have greater muscle retention with age, whereas endurance-trained older adults (i.e., 70 ± 0.7 years) had similar SMM as age-matched controls, and reduced SMM compared with younger sedentary (28 ± 0.1 years) or endurance-trained individuals [16]. Another cross-sectional study found that older (65 ± 8.5 years) adults who reported that they engaged in vigorous physical (50th percentile) activity had a 72% lower risk for having at least one criteria for sarcopenia [17]. In contrast, a study which observed forty high-level recreational Masters (i.e., 40–81 years) athletes who trained 4–5 times a week found, via magnetic resonance imaging (MRI), that mid-thigh muscle area and quadriceps area did not decline with progressing age [18]. The inconsistencies between these results may be due to these studies not measuring any other lifestyle factors that may lead to declines in SMM. For example, a cross-sectional study by ten Haaf et al. [19] found in a cohort of physically active (mean ± SD: 85 ± 53 metabolic equivalents (MET)/week) older (70 ± 4 years) adults that over 50% of participants did not meet the protein requirements of 1.2 g/kg BM/day as suggested for active older adults. Furthermore, Nilsson et al. [20] found that older community-dwelling women (67 ± 1.8 years) that habitually consumed ≥ 1.1 g/kg BM/day seemed to have an additional benefit on physical function outcomes when compared with those meeting the recommended daily allowance (e.g., 0.8 g/kg BM/day). Therefore, considering habitual daily protein intake for active older adults is higher (≥ 1.2 g/kg BM/day) than what is currently suggested for older adults, active older adults may be at an increased risk of muscle decline and strength due to the increased requirements for amino acid (AA) utilisation coupled with the progressive degeneration of SMM associated with ageing [21–23]. Therefore, determining whether there is an association between adequate protein intakes suggested for older adults (e.g., 1.2 g/kg BM/day) has on outcomes of sarcopenia should be explored.

The importance of maintaining strength throughout advancing age is highlighted by the recent shift in the European Working Group of Sarcopenia (EWGOSP2) clinical diagnosis that placed low handgrip strength at the forefront of their criteria [5]. Strength is defined as the ability to generate force, usually through the application of concentric muscle action [24]. Lower limb strength is associated with walking performance, the ability to get in and out of chair, step-climbing speed, and rate of falls [25]. Additionally, the correlation between handgrip strength (HGS) and isometric knee extensor strength allows grip strength to be used as a measure of whole body strength in sedentary older adults [26]. However, a study that looked at the ratio of HGS to quadriceps strength and found it to be significantly lower in the older group compared with the younger group indicating a greater age-related decline in quadriceps strength than HGS [27]. It is unknown whether these findings would be seen in a cohort of active older adults. Given these research inconsistencies and confounding factors that have not been adequately controlled for in previous research, it would be of interest to include a measure of upper and lower body strength outcomes for active older adults.

Along with declines in skeletal muscle size and strength, declines in skeletal muscle power and cardiorespiratory fitness have also been evident to occur with ageing [28, 29]. In a cross-sectional study of one-hundred community-dwelling older adults (65–89 years), skeletal muscle power was reported to decline in men at a rate of 3.5% per year [30]. Additionally, a study of Masters power athletes (i.e., sprinting, throwing, and jumping), aged 35–95 years, found skeletal muscle power dropped 1.2% per year, beginning from the age of 70 years [31]. The events that involved mostly upper limbs (e.g., shot put and javelin throw), showed a highest rate of decline (1.4% per year), compared with lower limb events (e.g., long jump decreased 1.1%). Additionally, there has been an observed decrease in $\dot{V}{\mathrm{O}}_{2\max }$ of 2.2 ml/kg/min (5.5%) per decade in Masters endurance-trained athletes [32]. However, this decline was significantly less than age-matched sedentary controls which had an observed decline of 3.3 ml/kg/min (12% per decade). It appears that regular physical activity during older age may play a role in being protective against outcomes related to sarcopenia in comparison to inactive older adults. Measures of muscle power and cardiorespiratory fitness may provide more reliable indicators of declines in muscle function in older populations that are considered ‘*active’* [12]. Moreover, another major limitation of many previous studies is the relatively few outcomes measured detecting intrinsic (e.g., inflammatory and anabolic resistance) [33, 34] and/or extrinsic (i.e., lifestyle factors) [35] factors, which makes it unclear as to whether these potential factors may contribute to the skeletal muscle functional decline, than ageing alone, in active older adults.

To our knowledge, the potential single and combined associations between the influences of dietary intake (e.g., protein intake) and exercise status in relation to the outcomes of SMM, strength, and power have not been investigated in a homogenous sample of ‘*active older adults*’ (≥ 50 years). While ≥ 50 years is not considered ‘older’ in the general spectrum of sarcopenia research, it is the age at which declines in SMM and strength begins to be noticeable. Therefore, the present study aimed to explore the link between age, physical activity level, dietary protein intake, and biological sex with SMM, strength, power, and physical capacity/ performance in active older adults (≥ 50 years).

## Subjects and Methods

### Study Population

**Fig. 1:**
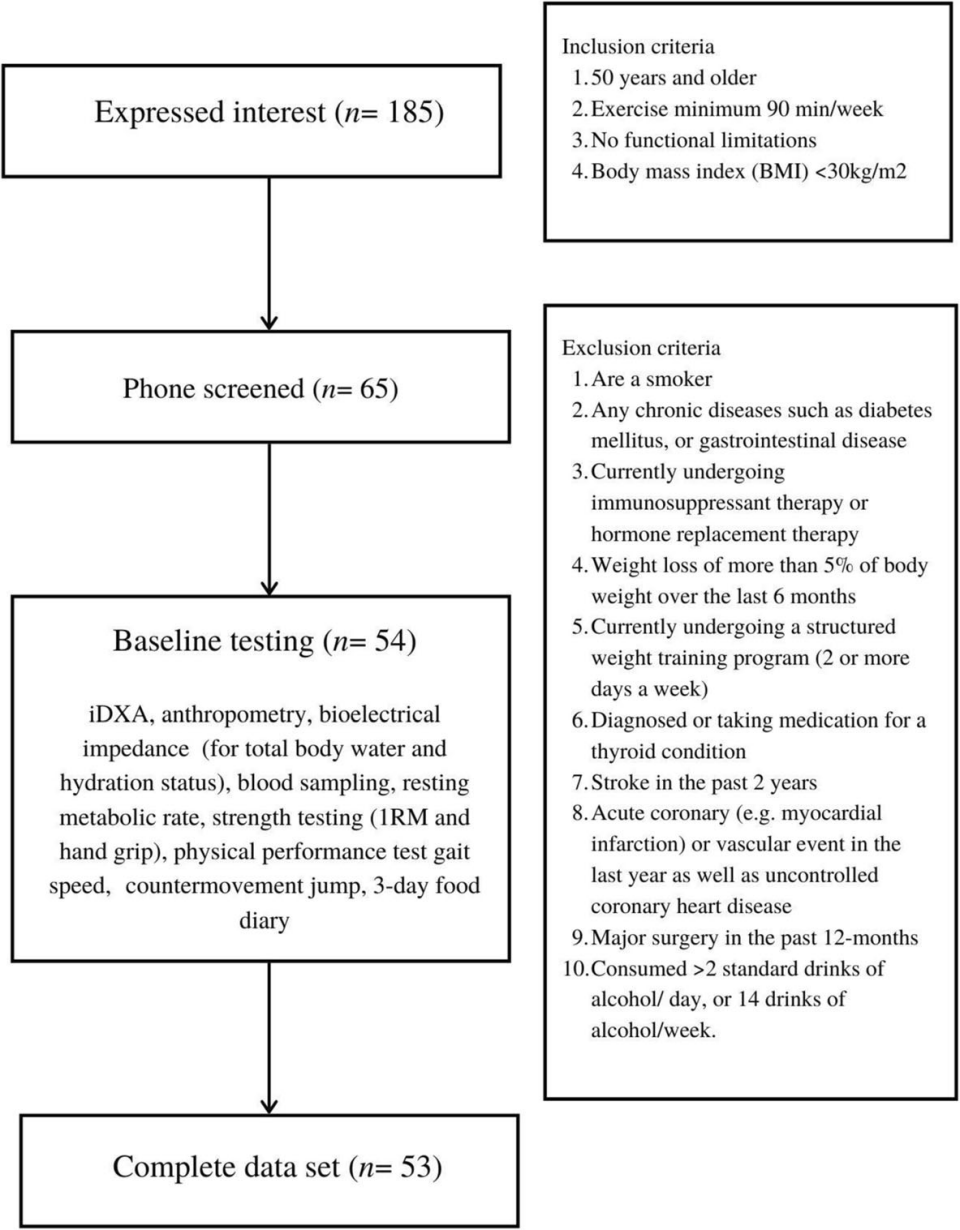
Flow diagram for the identifications, screening, eligibility, and participant completion

Fifty-four active middle-aged (mean ± SD: age 53.3 ± 2.8 years, BM 74.7 ± 15.2 kg, height 1.72 ± 0.10 m) and older adults (mean ± SD: age 64.6 ± 4.3 years, BM 74.5 ± 14.1 kg, height 1.72 ± 0.10 m) volunteered to participate in the study. Participants were eligible if they were ≥ 50 years, performed exercise training for recreational fitness and/or sports competitions ≥ 3/week, for ≥ 90 min/week, had no functional limitations, were free from chronic disease/disorders, were not taking medications that could interfere with SMM structure and/or function (e.g., corticosteroids, testosterone replacement, or anabolic drugs), were not undergoing immunosuppressive therapy or hormone replacement therapy, and were not adhering to structured resistance training program/s. All participants gave written informed consent. The study protocol obtained approval from the local ethics committee (Project number 12812), and the study was performed in accordance with the standards of ethics outlined in the Declaration of Helsinki. Data were collected during the period of September 2018 to January 2020, See Fig. 1 for participant flow.

### Exercise Volume

Prior to commencing any physical activity, participants filled out a physical activity readiness questionnaire (PAR-Q). They self-reported their level of physical activity including exercise intensity and volume per week, and the modality of exercise. Participants self-reported their level of activity including exercise volume (e.g., less than once per month, once per month, 2–3 times per week, 4–5 times per week, or > 5 times per week), intensity (e.g., vigorous, moderate and light), the time of exercise spent in each intensity and modality of training. All participants were categorised into low (≤ 149 min/week), moderate (≥ 150–299 min/week) or high (≥ 300 min/week) exercise volume [7].

### Dietary Assessment

Participants were educated and asked to complete a 3-day food–fluid diary prior to their initial visit. They were required to record all the foods and fluids on each main meal (breakfast, lunch and dinner) and any additional snacks (e.g., morning tea, afternoon tea, supper) ingested on 2 weekdays (Monday–Friday) and 1 day on the weekend (Saturday/Sunday) that most reflected their usual intake. Participants were required to specify the food and beverage quantities (e.g., grams, millilitres, litres, portions) and qualities (e.g., cooking method, brands of foods/beverages, types of foods/beverages). Data were analysed for outliers using boxplot. Identified outliers were removed prior to comparative data analysis procedures. Food–fluid diaries were analysed using FoodWorks v10.0 nutritional analysis software (Xyris Software, Brisbane, Australia, 2019) based on Australian food composition tables from Australian Food Composition Database (AFCD) 2019. Based on preliminary data, participants were then categorised into low (< 0.8 g/kg BM/day), moderate (0.8–1.19 g/kg BM/day), and high (≥ 1.2 g/kgB M/day) protein intake to assess the effects that daily dietary protein intake alone has on outcomes of SMM, strength, power, and physical performance markers [21].

### Experimental Procedure and Measurement of Outcomes

For measurements of outcome variables, participants were required to attend the laboratory for the period between 07.00 a.m. to 09.00 a.m. in a fasted and euhydrated state (296 ± 5.6 mOsmol/kg; 53.3 ± 6.4% TBW; Seca 515 MBCA, Seca Group, Hamburg, Germany), and after avoiding strenuous exercise for a 24-h period. All measurements were performed in the same order for all participants. Height was assessed using a fixed stadiometer (Holtain, Crosswell, Crymych, UK). BM was measured (Seca 515 MBCA) to the nearest 0.1 kg, using standardised anthropometrical procedures. Total (kg) and relative (%) FM and FFM, and bone mineral content were assessed by a trained radiographer using a dual-energy X-ray absorptiometry (Prodigy, GE Lunar, Madison, WI; with analysis software 14.10). Appendicular lean mass (ALM) was determined by adding the total arm and trunk lean mass and then it was adjusted for height (ALM/height^2^). Resting metabolic rate (RMR) was determined by indirect calorimeter (Vmax Encore Metabolic Cart; Carefusion, San Diego, CA) in temperate ambient conditions (22.2 ± 1.4 °C), and in accordance with best practice guidelines [36]. To comply with ethical procedures, prior to commencing the strength, power, and performance measures, participants were provided with a standardised breakfast (1.4 MJ, 15.3 g protein, 51.7 g carbohydrates, 6.8 g total fat). Physical assessment measures commenced ~ 30 min thereafter.

### Blood Collection and Analysis

Blood glucose concentration, haemoglobin, total and differential leukocyte counts (i.e., neutrophils, lymphocytes, and monocytes) were determined by HemoCue system (Glucose 201+, Hb201, and WBC DIFF, respectively; HemoCue AB, Ängelholm, Sweden) in duplicate from heparin whole blood samples. Coefficient of variation (CV) for blood glucose concentration, haemogobin and leukocyte counts were 4.1%, 1.8% and 13.6%, respectively.

The remaining heparin whole blood samples were centrifuged at 4000 rpm for 10 min within 15 min of sample collection. Aliquots of heparin plasma were placed in 1.5-ml microstorage tubes and frozen at – 80 °C until analysis, except x2 μl plasma was used to determine P_Osmol_ in duplicate (CV 1.0%), using a freeze point osmometry (Osmomat 030; Gonotec, Berlin, Germany).

Circulating concentrations of cortisol (DiaMetra, Perugia, Italy), insulin-like growth factor-1 (IGF-1) (Crux Biolab, Scoresby, Australia), insulin (Crux Biolab, Scoresby, Australia), testosterone (17b-OH-4-androstene-3-one; DiaMetra, Perugia, Italy), estradiol (17β-Estradiol; DiaMetra, Perugia, Italy) were measured by enzyme-linked immunosorbent assay (ELISA). Plasma concentrations of interleukin (IL)-2, IL-6, IL-1β, tumor necrosis factor (TNF)-α, IL-8, and IL-10 were determined by high sensitivity multiplex ELISA (HCYTOMAG-28SK; EMD Millipore, Darmstadt, Germany). All assays were performed as per manufacturer’s specifications, with standards and controls on each plate. The CV for analysed circulating biomarkers was ≤ 7.2%, and for systemic inflammatory cytokines was ≤ 13.5%.

### Strength Outcomes

Strength was assessed by performing a 1 repetition maximal strength (1-RM) in accordance with previously described procedures [37]. During a familiarisation trial, proper lifting technique was demonstrated, then participants were familiarised with each resistance machine (Hammer strength; LifeFitness, Sydney, Australia) by performing 8–10 repetitions of a light load (~ 50% of predicted 1-RM). After the successful completion of a further five to six repetitions at a heavier weight selected by the instructor, the workload was increased incrementally until only one repetition with correct technique could be completed. Participants were given 3–5 min rest in between attempts [38]. The value indicative of 1-RM was the highest load that could be raised in one single repetition using correct technique for leg press, *latissimus dorsi* (lat) pull down, and bench press. The 1-RMs were normalised by BM (1-RM/BM). Handgrip strength (HGS) was measured using a digital hand dynamometer (Jamar® Plus+ Digital hand dynamometer; Sammons Preston, Bolingbrook, IL, USA). HGS was measured in a standing position with the participants elbow by their side and flexed to a 90° angle and a neutral wrist position. Participants were asked to apply the maximum grip strength three times with both left and right hands; HGS was defined as the highest value for their dominant hand [39].

### Submaximal Incremental Bike Test

Submaximal aerobic fitness was determined using an incremental bike test using a cycle ergometer (*Corival,* Lode, Groningen, Netherlands) and a metabolic cart (Vmax Encore Metabolic Cart; Carefusion, San Diego, CA). The initial workload began at 1 watt (W) per kilogram of fat-free mass (W/kgFFM) and increased by 0.5 W/kgFFM every 3 min until participants could not maintain the speed at 60 RPM or higher or they reached a rating of perceived exertion (RPE) of 15–17 on the Borg scale [40]. Heart rate (HR) (Polar Electro, Kempele, Finland), $\dot{V}{\mathrm{O}}_{2\max }$, respiratory exchange ratio (RER), and RPE were measured every 3 min in real time. Cardiorespiratory fitness was expressed as watts/RER. Procedures were adjusted from standard fitness testing procedures [41].

### Countermovement Jump

A Force plate (400s+ Performance Force plate; Fitness Technology, Adelaide, Australia) was used to measure relative muscle power (W/kg), jump height (cm) and velocity (m/s) during a countermovement jump test (CMJ). Participants were asked to start in a full erect standing position in the middle of the force plate, then instructed to dip to a self-selected depth and “jump for maximal height”. Hands were kept on the hips to minimize any influence of arm swing [42]. Participants were asked to perform three attempts of a CMJ with 1-min rest in-between jumps. The Force plate was interfaced with computer software (Ballistic Measurement System; Fitness Technology, Adelaide, Australia), where the mean of three jumps was selected for further analysis.

### Gait Speed Measurement

To assess gait speed, a walking course of 4 m long was marked on the floor. The participant was instructed to walk from one end of the course to the other at their usual walking pace. The timer began as the participant started walking, and the timer was stopped with the first footfall after the 4-m line. The test was repeated twice and the average (of two scores) was determined. Gait speed was reported at seconds/meter.

### Statistical Analysis

Data in text and tables are presented as either mean ± SD (descriptive experimental data) or mean and 95% confidence interval (CI) (primary and secondary variables), where indicated. All statistical analyses were performed using IBM SPSS statistics software (Verson 25.0, IBM Corp, Armonk, NY). Prior to analysis, assumptions of normality in the data were made using Shapiro-Wilk test and visualisations of normality plots. Variables with multiple groups were examined using a general linear model (ANCOVA) or non-parametric ranked repeated measures, where appropriate. A Tukey’s post hoc test was applied to determine between group differences. In addition, adjustments for biological sex were performed. Variables with singular points were examined using independent *t* tests, or non-parametric Mann-Whitney *U* test. Significance was accepted at *p ≤* 0.05. Additionally, Cohen’s *d* was applied to determine the magnitude of effect size for significance differences, with *d* ≥ 0.20 for small, *d* ≥ .50 for medium, and *d* ≥ 0.80 for large effect size.

## Results

Table 1 presents the participant characteristics. Of the fifty-four participants included in the data collection, 53 were included in the analysis, due to a missing food diary (Fig. 1). Of the 53 participants, 86% were Caucasian, 10% were Asian, and 4% were southeast Asian. The participants from this study came from a variety of sporting backgrounds that were composed of endurance runners and race walkers (61%), cyclists (9%), aerobic gym goers (16%), or a combination of multiple activities (14%). Based on the EWGOS2 clinical diagnosis 6% (*n* = 3) had low HGS and were considered to have probable sarcopenia [5].

**Table 1:** Participant characteristics.

Characteristics	Values
Age, years	58.8 (57.0 to 61.0)
Height, m	1.71 (1.70 to 1.75)
Body mass (BM), kg	74.3 (70.2 to 78.3)
BMI, kg/m^2^	24.8 (22.0 to 26.0)
RMR, MJ/day	5.8 (5.5 to 6.1)
**EWOSP2 category for sarcopenia**	
Handgrip, (dominant), kg	37.0 (33.8 to 40.1)
ALM/ht^2^	7.7 (7.3 to 8.1)
Gait speed, m/sec	0.8 (0.8 to 0.9)
*n*, considered sarcopenic	0
**Exercise volume,**	
min/week	226 (191 to 260)
**Dietary protein intake,**	
g/kg BM/day	1.4 (1.2 to 1.5)
**iDXA measurements**	
FFM, kg	53.6 (50.6 to 56.7)
FM, %	28.4 (26.0 to 31.0)
Arm lean mass, kg	5.6 (5.1 to 6.1)
Leg lean mass, kg	17.5 (16.4 to 18.6)
**Strength, power, and physical performance**	
Leg press, kg/BM	1.9 (1.8 to 2.1)
Lateral pull down, kg/BM	1.0 (0.9 to 1.1)
Chest press, kg/BM	0.8 (0.7 to 0.9)
**Vertical jump**	
Jump height, cm	17.3 (3.0 to 28.0)
Relative power, W	29.5 (28.0 to 31.1)
Velocity, m/sec	1.9 (1.8 to 2.0)
**Cardiorespiratory fitness**	
Watts/RER,	111 (97 to 127)
**Biochemistry**	
BGL, mMol/L	4.8 (4.6 to 5.1)
Insulin, ulU/ml	7.1 (3.2 to 20.5)
IGF-1, pg/ml	97 (33 to 160)
Testosterone, ng/ml	1.4 (0.0 to 5.0)
Estradiol, pg/ml	26 (0 to 273)
Cortisol, nmol/L	366 (15 to 964)
WBC, pg/ml	4.8 (2.3 to 7.1)
Neutrophils, pg/ml	2.7 (1.1 to 4.5)
Lymphocytes, pg/ml	2.1 (1.0 to 3.9)
Monocytes, pg/ml	0.3 (0.1 to 1.1)
IL-2, pg/ml	4.1 (0.3 to 10.8)
IL-6, pg/ml	5.9 (0.8 to 36.8)
IL-8, pg/ml	5.9 (0.2 to 28.1)
IL-10, pg/ml	18.5 (1.0 to 50.1)
TNF-α, pg/ml	2.3 (0.4 to 5.6)

Mean (95% CI). *Abbreviations: ALM* appendicular muscle mass, *BGL* blood glucose levels, *BMI* body mass index, *BM* body mass, *EWSOP2* European Working Group of Sarcopenia 2, *FFM* fat-free mass, *FM* fat mass, *IGF* insulin-like growth factor, *IL* interleukin, *RER* respiratory exchange ratio, *RMR* resting metabolic rate, *TNF-α* tumour necrosis factor alpha, *W* watts

### Body Composition Stratified by Age, Exercise Volume, Dietary Protein Intake, and Biological Sex

The middle-aged group had a higher average bone mineral density compared with the older group, with a moderate association on age 7.6%, *d =* 0.562 (Table 2, A). Table 2 (B) shows between-group differences of outcomes based on the level of reported exercise volume; there was a trend towards significance with the low training group having 10.5%, and 9.5% higher FM compared with the moderate and high exercise volume groups, respectively. A significant difference was observed between total daily protein intake with BM, FM, and ALM/ht^2^ (Table 2, C). The high protein group (> 1.2 g/kg BM/day) weighed − 16.1kg (*p =* .025, *d* = 0.386) less than the low protein group. Table 2 (*D*) presents the differences in body composition based on biological sex. Male participants were 16 kg heavier than female participants (*d =* 1.30), had 28% higher FFM (*d =* 2.34), and showed higher arm (*d =* 3.01) and leg lean muscle mass (*d =* 2.44). Male participant appendicular muscle mass (ALM/ht^2^) was also significantly higher than female participants (*d =* 2.85). Female participants had significantly higher FM compared with male participants (*d =* 0.921).

**Table 2:** Body composition of participants based on age (A), exercise volume (B), protein intake (C), and biological sex (D)

	BM (kg)	FFM (kg)	FM (%)	Arm lean mass (kg)	Leg lean mass (kg)	BMD	ALM/ht^**2**^
**A.**							
Middle *n* = 26	74.7 (68.5 to 81.0)	55.3 (51.3 to 59.2)	27.0 (23.5 to 30.6)	5.6 (5.0 to 6.3)	18.0 (16.4 to 19.7)	1.3 (1.2 to 1.3)	7.8 (7.3 to 8.4)
Older *n* = 27	74.5 (69.2 to 80.3)	51.9 (47.3 to 56.6)	30.0 (26.2 to 34.0)	5.7 (5.1 to 6.4)	17.7 (16.1 to 19.2)	1.2 (1.1 to 1.2)	7.6 (7.0 to 8.1)
***p*** **value**	.936	.573	.250	.822	.459	.040	.451
**B.**							
Low *n* = 15	78.0 (70.0 to 86.1)	52.0 (45.4 to 58.3)	35.6 (31.2 to 40.0)	5.5 (4.5 to 6.5)	16.9 (14.7 to 19.1)	1.1 (1.1 to 1.3)	7.7 (6.9 to 8.6)
Moderate *n* = 24	75.8 (70.0 to 82.0)	56.4 (52.0 to 60.7)	25.1 (22.3 to 30.0)	5.8 (5.2 to 6.5)	18.6 (16.7 to 20.3)	1.3 (1.2 to 1.3)	7.8 (7.3 to 8.4)
High *n* = 14	69.5 (60.3 to 79.0)	51.0 (44.7 to 56.7)	26.1 (21.8 to 31.0)	5.4 (4.4 to 6.3)	16.7 (14.2 to 19.1)	1.2 (1.1 to 1.3)	7.5 (6.7 to 8.3)
***p*** **value**	.385	.405	.077	.344	.685	.916	.198
**C.**							
Low *n* = 7	86.1 (70.1 to 102.1)	57.1 (46.8 to 67.6)	35.2 (27.8 to 39.0)	6.7 (4.4 to 9.0)	19.7 (15.5 to 24.0)	1.2 (1.2 to 1.3)	8.6 (7.3 to 10.0)
Moderate *n* = 13	80.6 (72.6 to 92.0)	57.1 (50.7 to 63.5)	31.6 (25.9 to 37.3)	5.4 (4.6 to 6.3)	17.6 (14.6 to 20.6)	1.3 (1.1 to 1.4)	7.6 (6.6 to 8.6)
High *n =* 33	70.0^a^ (65.8 to 71.0)	51.8 (47.9 to 55.8)	25.3^a^ (22.2 to 28.3)	5.4 (4.8 to 6.0)	17.1 (15.7 to 18.4)	1.2 (1.1 to 1.3)	7.6 (7.1 to 8.0)
***p*** **value**	.025	.933	.005	.063	.263	.093	.143
**D.**							
Males *n* = 36	80.2 (75.5 to 85.0)	59.2 (56.3 to 62.0)	25.8 (23.1 to 28.4)	6.5 (6.2 to 6.9)	19.8 (19.7 to 20.8)	1.3 (1.2 to 1.3)	8.4 (8.0 to 8.7)
Females *n* = 17	64.1 (55.0 to 62.0)	42.4 (39.5 to 45.3)	34.1 (29.2 to 38.5)	3.8 (3.5 to 4.1)	13.4 (12.6 to 14.2)	1.1 (1.0 to 1.1)	6.5 (6.1 to 7.0)
***p*** **value**	< .001	< .001	< .001	*<* .001	< .001	< .001	< .001

Mean (95% CI). (A) Age: middle-age (50–59 years) and older (≥ 60 years). (B) Exercise volume: low (≥ 90–149 min), moderate (≥150–299 min), high (≥ 300 min). (C) Protein intake: low (≤ 0.8 g/kg BM/day), moderate (≥ 0.8–1.19 g/kg BM/ day), high (≥ 1.2 g/kg BM/day). (D) Males and females. Between group differences: ^a^ *p* < .05 vs low. *Abbreviations: ALM* appendicular muscle mass, *BM* body mass, *FFM* fat-free mass, *FM* fat mass, *BMD* bone mineral density

### Strength outcomes stratified by age, exercise volume, dietary protein intake, and biological sex

Significantly higher leg press (*d* = 0.758), *lat* pull down (*d =* 0.532), and chest press (*d =* 0.600) 1-RMs were observed in middle-aged compared with and older participants (Table 3*, A*). There was no substantial difference between HGS based on age. Based on exercise volume, there was a trend towards significance for 1-RM leg press, with post hoc analysis indicating a significant difference between groups for low vs. high training volumes (27%, *p =* .012, *d* = 0.902) and low vs. moderate (23%, *p =* .018, *d* = 1.06) exercise groups (Table 3*, B*). There was no significant differences observed for outcomes of strength based on daily protein intake (Table 3*, C*)**.** Male participants presented significantly higher relative 1-RM assessment compared with female participants (Table 3*, D)*. Leg press 1-RM was 20% greater in male compared with female participants (*d =* 0.708). Male participants showed 30–33% higher chest press (*d =* 1.42) and *lat* pull down (*d =* 0.400), and HGS (*d =* 2.39), compared with female participants.

**Table 3:** Strength outcomes stratified by age (A), exercise volume (B), protein intake (C), and biological sex (D)

	Leg press (kg/BM)	Chest press (kg/BM)	Lat pull down (kg/BM)	Handgrip, dominant (kg)
**A.**				
Middle *n* = 26	2.2 (2.0 to 2.3)	0.8 (0.8 to 1.0)	1.0 (0.9 to 1.3)	38.7 (34.1 to 43.0)
Older *n* = 27	1.8 (1.5 to 2.0)	0.7 (0.6 to 0.8)	0.9 (0.8 to 1.0)	37.3 (33.5 to 41.0)
***p*** **value**	.008	.042	.047	.264
**B.**				
Low *n* = 15	1.6 (1.3 to 1.9)	0.6 (0.5 to 0.8)	0.9 (0.7 to 1.0)	34.4 (27.7 to 41.0)
Moderate *n* = 24	2.1 (1.9 to 2.4)	0.8 (0.7 to 0.9)	1.0 (0.9 to 1.0)	40.5(36.3 to 44.6)
High *n* = 14	2.2 (1.9 to 2.4)	0.8 (0.7 to 1.0)	1.0 (0.9 to 1.1)	37.0 (28.3 to 39.7)
***p*** **value**	.051	.195	.289	.351
**C.**				
Low *n* = 8	1.8 (1.4 to 2.1)	0.7 (0.6 to 0.9)	0.9 (0.8 to 1.1)	44.3 (35.8 to 52.8)
Moderate *n* = 12	1.7 (1.4 to 2.1)	0.8 (0.6 to 1.0)	0.9 (0.7 to 1.0)	36.5 (29.4 to 43.7)
High *n* = 33	2.1 (1.9 to 2.3)	0.8 (0.7 to 0.9)	1.0 (0.9 to 1.1)	36.4 (32.7 to 40.1)
***p*** **value**	.053	.232	.289	.239
**D.**				
Males *n* = 36	2.1 (1.9 to 2.2)	0.9 (0.8 to 0.9)	1.1 (1.0 to 1.1)	42.6 (40.0 to 45.3)
Females *n* = 17	1.7 (1.4 to 2.0)	0.6 (0.5 to 0.7)	0.7 (0.7 to 0.8)	25.8 (23.0 to 29.0)
***p*** **value**	.011	< .001	< .001	< .001

Mean (95% CI). (A) Age: middle-age (50–59 years) and older (≥ 60 years). (B) Exercise volume: low (≥ 90–149 min), moderate (≥ 150–299 min), high (≥ 300 min). (C) Protein intake: low (≤ 0.8 g/kg BM/day), moderate (≥ 0.8–1.19 g/kg BM/ day), high (≥ 1.2 g/kg BM/day). (D) Males and females. *Abbreviations: BM* body mass

### Power and Physical Performance Outcomes Stratified by Age, Exercise Volume, Dietary Protein Intake, and Biological Sex

Comparing age to physical performance and power outcomes (Table 4*, A*)**,** the middle-aged group presented 16% higher relative power (W/kg) (*d =* 0.900) and 10% higher velocity (*d =* 0.755) compared to the older-aged group. Additionally, the middle-aged group, showed 30% higher average watts/RER, reflecting a higher cardiorespiratory fitness, compared with the older-aged group (*d =* 0.822). Moreover, the middle-aged group jumped on average 6 cm higher compared with the older group (*d =* 1.39). Exercise volume did not influence any substantial difference on any of the physical performance outcomes (Table 4*, B*). Comparing groups based on relative dietary protein intake, there was no significant difference between groups (Table 4*, C*). The comparison of power and physical performance outcomes based on biological sex (Table 4*, D*), indicated that male participants jumped 6 cm higher (*d =* 0.802), had 11% higher relative power (*d* = 0.721), and 13% higher velocity (*d* = 1.11) compared with female participants. There was no statistically significant difference between biological sex for gait speed and cardiorespiratory fitness.

**Table 4:** Performance and power outcomes stratified by age (A), training volume (B), protein intake (C), and biological sex (D)

	Jump height (cm)	Relative power (W/kg)	Velocity (m/s)	Gait speed (m/s)	Cardiorespiratory fitness (Watts/RER)
**A.**					
Middle *n* = 26	20.3 (18.5 to 22.1)	32.0 (30.0 to 33.6)	2.0 (0.0 to 2.0)	0.8 (0.7 to 0.8)	136 (119 to 533)
Older *n* = 27	14.5 (12.5 to 16.5)	26.4 (24.0 to 29.0)	1.8 (1.6 to 1.9)	0.8 (0.7 to 0.8)	85 (65 to 106)
***p*** **value**	< .001	.002	.006	.249	.004
**B.**					
Low *n* = 15	14.9 (11.5 to 18.4)	26.0 (22.7 to 29.1)	1.7 (1.6 to 1.9)	0.9 (0.8 to 0.9)	89 (64 to 118)
Moderate *n* = 24	18.8 (16.7 to 21.0)	31.3 (28.8 to 33.7)	2.0 (1.9 to 2.1)	0.8 (0.7 to 0.9)	129 (107 to 151)
High *n* = 14	17.3 (14.4 to 20.3)	29.2 (27.6 to 31.0)	1.9 (1.7 to 2.0)	0.8 (0.7 to 0.9)	114 (82 to 146)
***p*** **value**	.660	.275	.279	.271	.092
**C.**					
Low *n* = 8	16.3 (11.2 to 21.3)	27.3 (23.0 to 31.6)	1.9 (1.6 to 2.1)	0.8 (0.7 to 0.9)	107 (65 to 149)
Moderate *n* = 12	17.8 (13.7 to 22.0)	26.0 (19.5 to 32.6)	1.7 (1.4 to 2.1)	0.8 (0.7 to 0.8)	117 (98 to 138)
High *n =* 33	17.6 (16.0 to 19.1)	31.2 (29.6 to 33.0)	2.0 (1.9 to 2.1)	0.8 (0.8 to 0.9)	117 (100 to 129)
***p*** **value**	.580	.056	.138	.641	.408
**D.**					
Males *n* = 36	19.0 (17.1 to 21.0)	30.4 (28.4 to 32.5)	2.0 (1.9 to 2.1)	0.8 (0.7 to 0.8)	121 (103 to 139)
Females *n* = 17	14.1 (11.5 to 16.7)	26.9 (24.0 to 30.0)	1.7 (1.6 to 1.8)	0.8 (0.8 to 0.9)	98 (71 to 124)
***p*** **value**	.003	.048	.001	.611	.136

Mean (95% CI). (A) Age: middle-age (50–59 years) and older (≥ 60 years). (B) Exercise volume: low (≥ 90–149 min), moderate (≥ 150–299 min), high (≥ 300 min). (C) Protein intake: low (≤ 0.8g/kg BM/day), moderate (≥ 0.8–1.19g/kg BM/ day), high (≥ 1.2g/kg BM/day). (D) Males and females. Abbreviations: *RER* respiratory exchange ratio, *W* watts

### Systemic Hormonal and Inflammatory Cytokine Profiles

There were no main significant associations between systematic hormonal or inflammatory cytokine markers on outcomes related to age, training status, or protein intake (supplementary material 1-3). There was a significant difference in testosterone, blood glucose, insulin, resting neutrophils, and monocytes for biological sex (supplementary material 4).

## Discussion

This study aimed to assess the link between self-reported and objective measures of age, physical activity, habitual dietary protein intake, and biological sex as they relate to measures of body composition, strength, power, and physical performance outcomes in a population of active older adults. The main findings were (1) middle-aged and older adults had no significant differences in body composition, but did display differences in strength, power, and performance; (2) higher exercise volumes (≥ 150 min/week) in active older adults had a trend towards significance in leg strength and lower body fat; however, there were no other significant differences between any other outcomes; (3) higher dietary protein intakes (≥ 1.2 g/kg BM/day) was linked with lower FM and body weight compared with lower protein intakes (< 0.8 g/kg BM/day); and (4) significant differences in body composition, strength, and power outcomes exist between male and female active older participants. There are many cross-sectional studies that examine some of these outcome measures in either community dwellers or frail and institutionalised older adults [43]. However, the current study is the first to comprehensively explore these prospective relationships in a cohort of active older adults. Overall, this study demonstrated that the contributions of age, physical activity, daily dietary protein intake, and biological sex can help explain individual variation in outcomes related to changes in body composition, strength, power, and physical performance.

Based on the EWGSOP2 clinical diagnosis criteria, 6% of the participants had low HGS and were considered to have “probable” sarcopenia. However, when analysed further, these participants were not confirmed to have sarcopenia based on muscle quantity [5]. This prevalence for sarcopenia is substantially lower than the 1–29% reported rates for community-dwelling adults over the age of 50 years [44]. A similar study by Fien et al. [45] found that in a cohort of 156 Masters athletes, 3.8% were considered to be below the sarcopenic HGS cutoff points. The difference in results is likely due to the larger sample size and the higher level of training of the Masters athletes compared with the more recreationally active older adults used in the current study. Considering that the prevalence of sarcopenia in aged-care facilities have been reported as high as 41% [44], our data suggest that active middle-aged and older adults levels of SMM, skeletal muscular strength, and physical function place them at very low risk of adverse health effects caused by sarcopenia and ageing, and that even higher levels of training may mitigate this [46].

Ageing is often associated with a decline in FFM, strength, and physical performance that is accelerated with sedentary behaviour [47]. The effects on ageing alone on these outcomes in Masters or recreationally active adults, is less defined. In the current study, there was no observed differences in body composition based on age. However, for strength, skeletal muscle power (e.g., CMJ), and physical performance outcomes, the middle-aged group had ≥ 10% higher strength (i.e., 1-RM leg press, chest press, and lateral pull down), jumped 28% higher, and had 30% higher cardiorespiratory fitness compared with the older-aged group. The decline in muscle power with ageing has been indicated to decline more rapidly than SMM and muscular strength, and therefore muscle power may be more of a substantial indicator of physical function with ageing, especially in the active population [30]. A cross-sectional study by Pearson et al. [29] found that peak power (W) declined with increasing age at a similar rate between elite Masters weight lifters and controls (1.2% and 1.3% per year); while muscular strength declined at a similar but lower rate (0.6% and 0.5% per year). The cause of reduced force- and power-producing capabilities relative to muscle size with ageing appears to be attributed to a reduction in type II fast-twitch muscle fibre size. An original study by Lexell et al. [48] observed from cross-sections of the *vastus lateralis* in 30 male cadavers that older adults (71 ± 1 years) had on average 18% less muscle fibre size, and 25% decline in total muscle fibres compared with younger adults (30 ± 6 years). The difference in total muscle size was purposed to be accounted by a marked reduction in the number of myofibrils in the older muscle (478,000 vs 364,000). In support of this, a more recent study by Nilwik et al. [49] found that type II muscle fibres were substantially smaller (29%) in older (71 ± 1 years) versus a young (23 ± 1 years) population. The decrease in individual muscle fibres, particularly type II, could potentially explain the observed lower muscle strength and lower body power in the older-aged group compared with the middle-aged group in the current study. Additionally, in relation to age, there was a significant difference in cardiorespiratory fitness with the average fitness levels being 30% less in the older group than the middle-aged group. There have been numerous cross-sectional and longitudinal studies reporting a decrease in $\dot{V}{\mathrm{O}}_2$ max with age, irrespective of training status [32, 50]. These studies have reported a rate of decline in sedentary individuals to be approximately 10% per decade [28, 51], whilst even highly active individuals have a decline of ~ 5% [52–54]. A study by Stathokostas et al. [28] in a 10-year longitudinal study in healthy older adults (73.5 ± 6.4 years; 28 women, 72.1 ± 5.3 years) observed a 14% and 7% decline in *V*O_2max_ in men and women, respectively. Additional analysis showed that age-related changes in *V*O_2max_ were not significantly related to physical activity [28]. Irrespective of the mechanisms, this indicates that decreasing muscle power and strength and cardiorespiratory fitness in older age may be cause for concern, and it is clear that activity alone may not mitigate these changes occurring with age. Additionally, the maintenance of muscle power and cardiorespiratory fitness in older adults may be an important target for intervention, given its implications for ambulation and physical function in older adults.

The current recommendations based on the WHO suggest that all adults should engage in at least 150 min of moderate activity throughout the week to support bone, joint, and muscle health, which in turn may reduce functional limitations, prevent falls, and promote independent living [7]. There were no significant associations between exercise volume and any of the outcomes. However, there was a notable trend towards significance, with the results indicating that individuals who achieved the recommended exercise volume (≥ 150–299 min per week) or higher (≥ 300 min per week), when compared with the lower training group (≥ 90–149 min/week), had a trend towards a significant greater leg strength and lower body fat in those that achieved the recommended exercise volume or more. Considering that the low training group had the highest proportion of females (50%, supplementary material 2), and females typically present with ~ 10% higher body fat and generally produce less power and strength compared with men, this could explain the trend between the groups, more than training alone [55, 56]. It is likely that there was not a more significant difference in results as the participants within this cross-sectional study were considered ‘active’; this is highlighted by the fact that there were no significant differences in cardiorespiratory fitness (CRF) observed between groups. Furthermore, a cross-sectional study by Marcos-Pardo et al. [17] found that a measured sedentary time over 300 min/week found a significant association for increased risk factors for lower functional performance and failing at least one variable associated with sarcopenia. Inadequate physical activity levels have also been associated with an increase risk in chronic diseases (e.g., type II diabetes), obesity, and unfavourable cardiovascular markers [6, 57]. A limitation for this study is the lack of comparison made with a non-athletic control group. Therefore, despite there being a trend in body fat percentage and leg strength between the exercise groups, it is not known if these are any better than the general population and if the larger proportion of females were the cause of the significant difference.

Higher protein intakes (e.g., ≥ 1.2 g/kg BM/day) have been advocated for physically active older adults to overcome the increased requirements of AA utilisation due to exercising, alongside increased requirements caused by ageing [21]. This study found that higher intakes of protein (≥ 1.2 g/kg BM per day) were associated with lower body weight, lower fat mass compared with the moderate and low protein intake groups. While the effect size comparing the low and moderate intakes was small for the same variables (*d <* 0.5). Our findings partially support the assumption that physically active older adults may require higher amounts of protein (≥ 1.2 g/kg BM per day) than the current recommendations (0.8 g/kg BM per day) and may be a key factor in preventing the decline in muscle strength in older adults [58, 59]. There have been numerous observational studies that have correlated protein intake in relation to sarcopenia to explore diet–muscle health relationships [59]. However, the majority of these studies are conducted in free-living community dwellers, with limited studies in physically active older adults. One of the few studies in active older adults compared protein intakes and physical function in active (150 min per week of moderate-to-vigorous activity) older (67.5 ± 1.8 years) women and found a significant difference in self-reported physical function—as measured using handgrip strength and short physical performance battery (SPPB)—in women with higher protein intakes (> 1.1 g/kg BM per day) compared with low protein intakes (0.8 g/kg BM per day) [21]. Additionally, a recent systematic review and meta-analysis of observational studies assessing protein intake with various physical performance outcomes found that reasonably high (1.0 g/kg BM per day) and very high protein intakes (> 1.2 g/kg BM per day) were associated with more favourable lower-limb physical performance (*p <* .050) and lower-limb strength (*p =* .050) when compared with low protein (< 0.8 g/kg BM per day) in community-dwelling older adults [59]. Furthermore, after supplementation of protein for 12 weeks in physically active older adults who habitually consumed low protein (< 1.0 g/kg BM/day), there was a significant increase in physical performance and lean body mass, and a decrease in FM was observed after race walking training [60]. Physical activity increases the sensitivity of skeletal muscle to the anabolic properties of protein consumption, and the active older population, which exercises at higher amounts than the general population, requires more protein [6, 61]. However, total protein intake *per se* may not be sufficient enough to support active ageing. Numerous studies have indicated that distribution, timing, and quality of protein are important to consider [62]. Therefore, future studies should focus on these factors alongside total protein intake in an active older population.

The results of this study based on gender found that males had significantly more FMM, ALM, and regional muscle mass (arms and legs). The findings of this study strengthen the already abundant results of previous studies that have reported significant sex differences in lean muscle mass, as estimated by DEXA, MRI, and CT [56, 63, 64]. On average, FFM was 28% higher in males than in women with a large effect size. This gender difference remained after adjusting for height (ALM/ht^2^) where men had 25% more SMM compared with women. The muscle distribution between genders showed that women had 41% less arm SMM and 31% less leg SMM. These findings support previous works by Gallagher and Heymsfield [64], which reported that females have a larger proportion of their appendicular muscle mass in their lower extremities in comparsion to males (as estimated by DEXA). Additionally, Janssen et al. [63] reported similar gender differences in upper (40%) and lower body (33%) muscle mass based on MRI images. This difference in muscle distribution when adjusting for differences in total body mass is the likely cause for the observed differences in strength outcomes. For example, the observed gender differences in lower body strength (~ 30%) which are smaller than those observed for upper body strength (~ 50%). The differences in skeletal muscle mass, strength, and power between the genders are most likely due to the greater capacity for muscular hypertrophy as a result of higher levels of circulating testostrone seen in males [65]. This is highlighted in the differences in the biochemical parameters observed between biological sex (supplementary material 4), where males have 8 times greater levels of testostrone than females. Additionally, considering the clear physiological differences between genders, future intervention studies should consider differentiating between gender.

The declines in SMM and strength observed with increasing age may be due to the changes in systemic anabolic hormones (e.g., testosterone and IGF-1), and chronic low-grade inflammation (e.g., ‘*inflammaging’*) [65–68]. Inflammaging is characterised by a chronic low-grade systemic inflammatory cytokine response such as tumour necrosis factor-α (TNF-α) and interleukin (IL)-6, and a decrease in anti-inflammatory cytokine such as IL-10 [69, 70]. Additionally, there is an inverse dose relationship that has been observed in multiple observational studies between the level of physical activity and such systematic inflammatory biomarkers [71]. For example, Colbert et al. [72] observed in a large scale study (*n =* 2964) an inverse relationship between higher levels of self-reported physical activity (≥ 180 min/week) and inflammatory biomarkers including C-reactive protein (CRP) and IL-6 after adjusting for body fat. However, in the current study, there were no observed differences in any of the systemic anabolic hormones or inflammatory cytokines associated with age, training status, or protein intake (supplementary materals 1-3). Nonetheless, intervention studies have reported signficiant effects of exercise and reducing inflammatory biomarkers in the ageing population [71, 72]. For example, Kohut et al [73] reported a significant decrease in IL-18, CRP, and IL-6 in older adults (> 64 years) after performing aerobic exercise 3 days a week, for 45 min at 65–80% *V*O_2peak._ Therefore, considering that within the current study cohort they were considered active within participants’ habitual lifestyles, this healthy active older adult population may have been too similar to detect the modest group differences in systemic hormonal and/or inflammatory profiles seen in previous studies with greater participant variability.

The strengths of this current study is the use of comprehensive outcomes measured including FFM, strength, physical performance outcome taking into consideration more relevant markers for active ageing cohort (e.g., CMJ), and hormonal and inflammatory parameters. This is the first study to assess the contributions of age, physical activity, daily protein intake, and biological sex can explain the individual variation in outcomes related to changes in body composition, strength, power, and/or cardiorespiratory fitness in a cohort of active older adults. The current study included the use of a 3-day food diary to infer daily dietary intake patterns and self-reported physical activity to estimate total physical activity levels. While these are acknowledged to be potential limitations due to the nature of using self-reported measures, based on the reported intake of the same cohort in an intervention study [74], it was observed that the energy and protein intakes of the 3-day food diaries did not change in the control groups over the course of a 12-week trial. For example, average relative protein intakes were reported to be 1.6 g/kg BM/day at baseline and 6 weeks and 1.4 g/kg BM/day at 12 weeks without any significant differences. Therefore, considering that the 3-day food diary used was consistently shown to be similar over 12 weeks, it is likely to reflect their true food intake in the current study. Furthermore, the average self-reported physical activity levels of the current study were 228 min/week. In the same intervention study [74], when physical activity was objectively measured using an ActiGraph accelerometer for 12 weeks, the mean time in moderate (3.00–5.99 metabolic equivalents (METS)) physical activity was 200–317 min/day and mean time in vigorous (≥ 6 METs) physical activity was 16–21 min/day. Considering that the accelerometer measured all physical activity, including incidental activity, it is likely that the self-reported physical activity at baseline within this current study was indeed a reliable method of measurement and did not involve over reporting of physical activity, which is commonly observed in studies [75]. This is likely due to the inclusion of active individuals that engage in regular structured physical activity, therefore can more reliably recall habitual physical activity. Lastly, it is acknowledged that this current study is a relatively small sample size. However, we believe that this study may provide initial preliminary data to help contextualise future intervention trials and identifies methodological gaps in sarcopenic research in active older adults.

## Conclusion

This study showed that the contributions of age, physical activity, daily protein intake, and gender can help explain individual variation in outcomes related to changes in body composition, strength, power, and performance in a cohort of active older adults. Further comparisons indicated that this cohort is at a low risk of adverse outcomes caused by sarcopenia. Strength and power outcomes were influenced by age, training status, protein intake, and biological sex.

## Supplementary Information

**Additional file 1: Supplementary Material 1**. Supplementary material of participants based on age. **Supplementary Material 2**. Supplementary material of participants based on training volume. **Supplementary Material 3**. Supplementary material of participants based on daily protein intake **Supplementary Material 4**. Supplementary material of participants based on biological sex.

## Data Availability

The datasets used and/or analysed during the current study are available from the corresponding author on reasonable request.

## Abbreviations

1RM: 1 repetition maximal
ALM: Appendicular muscle
BGL: Blood glucose
BM: Body mass
BMD: Bone mineral density
CSA: Cross-sectional area
CMJ: Countermovement jump
CV: Coefficient of variation
EWOSP2: European Working Group of Sarcopenia
FFM: Fat-free mass
FM: Fat mass
HGS: Handgrip strength
HR: Heart rate
IGF-1: Insulin-like growth factor-1
IL: Interleukin
RER: Respiratory exchange ratio
RMR: Resting metabolic rate
RPE: Rating of perceived exertion
SMM: Skeletal muscle mass
TNF- α: Tumour necrosis factor alpha
W: Watts
