# Supplementary material for: Sarcopenic Characteristics of Active Older Adults: a Cross-Sectional Exploration
Source: Sports Med Open. 2021 May 17;7:32. doi: 10.1186/s40798-021-00323-9 (PMC8128944; doi:10.1186/s40798-021-00323-9)
Supplement: Supplementary file 1 — Additional file 1: Supplementary Material 1. Supplementary material of participants based on age. Supplementary Material 2. Supplementary material of participants based on training volume. Supplementary Material 3. Supplementary material of participants based on daily protein intake Supplementary Material 4. Supplementary material of participants based on biological sex. [file 40798_2021_323_MOESM1_ESM.docx]

**The effects of a high protein dairy milk beverage with or without progressive resistance training on fat-free mass, skeletal muscle strength and power, and functional performance in healthy active older adults: A 12-week randomized controlled trial.**

Zoya Huschtscha^1^, Alexandra Parr^1^, Judi Porter^1,2^, *Ricardo J.S Costa^1^

^1^ Monash University, Department of Nutrition Dietetics & Food, Level 1, 264 Ferntree Gully Road, Notting Hill, 3168 Victoria, Australia.

^2^ Deakin University, Institute for Physical Activity and Nutrition (IPAN), School of Exercise and Nutrition Sciences, Geelong, Australia

***Corresponding author:**

Ricardo J.S. Costa: Monash University, Department of Nutrition Dietetics & Food, Level 1, 264 Ferntree Gully Road, Notting Hill, 3168, Victoria, Australia. Telephone: 00 61 3 99024270. [ricardo.costa@monash.edu](mailto:ricardo.costa@monash.edu)

| **Supplementary Material 1.** Supplementary material of participants based on age. | | | |
| --- | --- | --- | --- |
|  | **Middle**  ***n*= 26** | **Older**  ***n*= 27** | ***p*-value** |
| Females, % | 37 | 22 |  |
| Age, years | 53.3 (52.2 to 54.5) | 64.6 (63.0 to 66.4) | .016 |
| Height, m | 1.72 (1.67 to 1.76) | 1.72 (1.67 to 1.76) | .926 |
| Weight, kg | 74.7 (68.5 to 81.0) | 74.5 (69.2 to 80.3) | .936 |
| BMI, kg/m^2^ | 24.7 (23.4 to 26.0) | 25.0 (24.0 to 26.2) | .788 |
| **Self-reported Exercise** |  |  |  |
| Ex volume, min/week | 228 (183 to 274) | 227 (172 to 283) | .979 |
| **Resting Metabolic Rate** |  |  |  |
| MJ/day | 6.0 (5.6 to 6.4) | 5.6 (5.1 to 6.0) | .177 |
| **Daily protein intake** |  |  |  |
| g/kg BM/day | 1.4 (1.2 to 1.5) | 1.3 (1.1 to 1.5) | .837 |
| **Biochemistry** |  |  |  |
| BGL, mmol/L | 4.9 (4.5 to 5.4) | 5.0 (4.8 to 5.3) | .195 |
| Insulin, ulU/ml | 7.5 (4.7 to 9.5) | 6.7 (5.8 to 8.0) | .450 |
| IGF-1, pg/ml | 72 (26 to 118) | 224 (61 to 509) | .366 |
| Testosterone, ng/ml | 1.0 (0.5 to 1.6) | 1.9 (1.2 to 2.6) | .457 |
| Estradiol, pg/ml | 30.5 (5.3 to 55.6) | 26.6 (3.6 to 49.5) | .683 |
| Cortisol, nmol/L | 361 (284 to 438) | 393 (297 to 488) | .615 |
| WBC x10^9^ | 4.8 (4.3 to 5.3) | 5.6 (5.0 to 6.2) | .037 |
| Neutrophils x10^9^ | 2.5 (2.2 to 3.0) | 3.0 (2.6 to 3.2) | .168 |
| Lymphocytes x10^9^ | 2.0 (1.7 to 2.2) | 2.1 (2.0 to 2.6) | .160 |
| Monocytes x10^9^ | 0.3 (0.2 to 0.4) | 0.3 (0.3 to 0.4) | .294 |
| IL-2, pg/ml | 4.6 (2.9 to 5.8) | 3.7 (2.8 to 4.6) | .531 |
| IL-6, pg/ml | 5.0 (0.9 to 8.3) | 6.7 (2.5 to 11.4) | .557 |
| IL-8, pg/ml | 5.7 (1.9 to 7.5) | 6.6 ( 3.3 to 10.0) | .742 |
| IL-10, pg/ml | 17.6 (11.0 to 25.6) | 19.4 (13.7 to 25.8) | .545 |
| TNF-α, pg/ml | 2.2 (1.3 to 2.7) | 2.3 (2.0 to 2.8) | .545 |
| Mean (95% CI). Age: middle-age (50-59 years) and older (≥60 years). **Abbreviations:** BGL: blood glucose levels, BM, body mass, BMI: body mass index, IGF: insulin-like growth factor, IL: interleukin, m, metres, MJ: Megajoules, WBC: white blood cells. | | | |

| **Supplementary Material 2.**  Supplementary material of participants based on training volume. | | | | |
| --- | --- | --- | --- | --- |
|  | **Low**  ***n*= 15** | **Moderate**  ***n*= 24** | **High**  ***n*= 14** | ***p-*value** |
| Females, % | 50 | 21 | 35 |  |
| Age, years | 60.1 (56.0 to 63.0) | 58.4 (55.6 to 62.3) | 59.0 (57.0 to 61.0) | 0.720 |
| Height, m | 1.72 (1.66 to 1.80) | 1.74 (1.70 to 1.80) | 1.7 (1.61 to 1.80) | .065 |
| Weight, kg | 78.0 (70.0 to 86.1) | 75.8 (70.0 to 82.0) | 69.5 (60.3 to 79.0) | .285 |
| BMI, kg/m^2^ | 25.3 (23.3 to 27.8) | 24.5 (23.3 to 26.0) | 24.1 (23.7 to 25.5) | .631 |
| **Daily protein intake** |  |  |  |  |
| g/kg BM/day | 1.2 (1.1 to 1.4) | 1.3 (1.1 to 1.5) | 1.5 (1.2 to 1.8) | .243 |
| **Resting metabolic rate** | |  |  |  |
| MJ/ day | 5.7 (5.1 to 6.3) | 6.0 (5.5 to 6.5) | 5.5 (5.0 to 6.0) | .160 |
| **Biochemistry** |  |  |  |  |
| BGL, mmol/L | 5.2 (4.8 to 5.6) | 4.9 (4.5 to 5.1) | 4.8 (4.5 to 5.2) | .251 |
| Insulin, ulU/ml | 6.6 (4.0 to 9.2) | 7.2 (5.2 to 9.2) | 7.1 (6.1 to 8.1) | .456 |
| IGF-1, pg/ml | 125 (46 to 204) | 155 (40 to 271) | 160 (64 to 385) | .985 |
| Testosterone, ng/ml | 1.0 (0.3 to 1.7) | 1.9 (1.3 to 2.5) | 1.2 (0.5 to 1.9) | .184 |
| Estradiol, pg/ml | 22.1 (14.0 to 58.2) | 32.5 (6.0 to 59.0) | 29.0 (4.0 to 53.0) | .813 |
| Cortisol, nmol/L | 353 (266 to 440) | 400 (294 to 507) | 364 (257 to 470) | .884 |
| Neutrophils x10^9^ | 2.8 (2.1 to 3.4) | 2.7 (2.3 to 3.1) | 2.5 (1.9 to 3.0) | .508 |
| Lymphocytes x10^9^ | 2.1 (1.7 to 2.3) | 1.9 (1.6 to 2.2) | 2.2 (1.7 to 2.6) | .864 |
| Monocytes x10^9^ | 0.3 (0.2 to 0.3) | 0.3 (0.3 to 0.4) | 0.3 (0.3 to 0.4) | .519 |
| IL-2, pg/ml | 3.8 (1.9 to 5.7) | 4.4 (3.0 to 5.0) | 4.3 (3.0 to 5.7) | .864 |
| IL-6, pg/ml | 5.9 (0.2 to 12.0) | 5.3 (1.2 to 9.4) | 6.7 (2.6 to 11.0) | .895 |
| IL-8, pg/ml | 5.9 (2.0 to 9.8) | 4.2 (2.0 to 6.1) | 9.0 (2.8 to 15.1) | .164 |
| IL-10, pg/ml | 20.6 (12.6 to 28.6) | 19.4 (13.2 to 25.7) | 14.3 (7.4 to 21.3) | .462 |
| TNF-α, pg/ml | 2.6 (1.7 to 3.5) | 2.2 (1.6 to 2.7) | 2.1 (1.5 to 2.7) | .464 |
| Mean (95% CI). Exercise volume: low (≥90-149 min), moderate (≥150-299 min), high (≥300 min). Between group differences: ^a^ *p*< .05 vs low.  **Abbreviations:** BGL: blood glucose levels, BM, body mass, BMI: body mass index, IGF: insulin-like growth factor, IL: interleukin, m, metres, MJ: Megajoules, WBC: white blood cells. | | | | |

| **Supplementary Material 3.** Supplementary material of participants based on daily protein intake | | | | |
| --- | --- | --- | --- | --- |
|  | **Low**  ***n*= 8** | **Moderate**  ***n*= 12** | **High**  ***n=* 33** | ***p-valuep*-value** |
| Females, %  Age, years | 0  61.8 (54.1 to 69.5) | 20  56.1 (53.2 to 59.0) | 50  56.6 (52.1 to 62.0) | .170 |
| Height, m | 1.70 (1.60 to 1.80) | 1.73 (1.67 to 1.80) | 1.71 (1.67 to 1.80) | .699 |
| Weight, kg | 86.1 (70.1 to 102.1) | 80.6 (72.6 to 92.0) | 70.0 (65.8 to 71.0)^a^ | .025 |
| BMI, kg/m^2^ | 27.5 (24.5 to 29.0) | 26.0 (23.4 to 28.4) | 23.5 (22.6 to 24.5)^a^ | .001 |
| **Self-reported exercise** |  |  |  |  |
| Ex volume, min/week | 200 (119 to 280) | 162 (108 to 217) | 258 (210 to 305) | .091 |
| **Resting metabolic rate** |  |  |  |  |
| MJ/day | 5.8 (4.7 to 7.0) | 6.0 (5.1 to 7.0) | 5.7 (5.4 to 6.1) | .450 |
| **Biochemistry** |  |  |  |  |
| BGL, mmol/L | 5.3 (4.7 to 5.9) | 4.8 (4.5 to 5.2) | 5.0 (4.8 to 5.2) | .402 |
| Insulin, ulU/ml | 7.6 (5.3 to 9.1) | 7.7 (4.8 to 10.5) | 6.7 (5.8 to 7.9) | .722 |
| IGF-1, pg/ml | 138 (50 to 228) | 136 (44 to 229) | 154 (75 to 410) | .976 |
| Testosterone, ng/ml | 1.5 (0.3 to 2.8) | 1.1 (0.4 to 1.7) | 1.7 (1.1 to 2.2) | .330 |
| Estradiol, pg/ml | 14.0 (1.8 to 26.1) | 21.8 (15.7 to 59.5) | 35.5 (12.7 to 58.4) | .560 |
| Cortisol, nmol/L | 313 (143 to 482) | 380 (277 to 483) | 374 (298 to 449) | .752 |
| Neutrophils x10^9^ | 2.9 (2.5 to 3.4) | 2.8 (2.3 to 2.9) | 2.6 (2.3 to 2.9) | .553 |
| Lymphocytes x10^9^ | 2.3 (1.6 to 2.9) | 2.3 (1.9 to 2.7) | 2.0 (1.8 to 2.3) | .238 |
| Monocytes x10^9^ | 0.3 (0.2 to 0.5) | 0.4 (0.2 to 0.5) | 0.3 (0.3 to 0.4) | .547 |
| IL-2, pg/ml | 3.2 (0.7 to 5.7) | 3.9 (2.6 to 5.2) | 4.4 (3.4 to 5.4) | .580 |
| IL-6, pg/ml | 1.5 (0.1 to 3.1) | 3.8 (0.8 to 6.8) | 7.5 (3.7 to 11.3) | .258 |
| IL-8, pg/ml | 1.9 (0.9 to 2.9) | 5.2 (0.7 to 9.5) | 7.1 (4.2 to 10.1) | .150 |
| IL-10, pg/ml | 17.0 (2.8 to 31.1) | 18.3 (11.8 to 24.8) | 18.8 (13.3 to 24.4) | .872 |
| TNF-α, pg/ml | 1.8 (1.7 to 2.6) | 2.6 (1.8 to 3.3) | 2.2 (1.7 to 2.7) | .475 |
| Mean (95% CI). Protein intake: low (<0.8 g/kg BM/day), Moderate (≥0.8-1.19 g/kg BM/day), high (≥1.2g/kg BM/day). Between group differences: ^a^ *p*< .05 vs low. **Abbreviations:** BGL: blood glucose levels, BM, body mass, BMI: body mass index, IGF: insulin-like growth factor, IL: interleukin, m, metres, MJ: Megajoules, WBC: white blood cells. | | | | |

| **Supplementary Material 4.**  Supplementary material of participants based on biological sex | | | |
| --- | --- | --- | --- |
|  | **Males**  ***n*= 36** | **Females**  ***n*= 17** | ***p-valuep*-value** |
| Age, years | 59.1 (57.0 to 61.4) | 58.3 (55.0 to 62.0) | .702 |
| Height, m | 1.77 (1.74 to 1.80) | 1.62 (1.60 to 1.65) | < .001 |
| Weight, kg | 80.2 (75.5 to 85.0) | 64.1 (55.0 to 62.0) | < .001 |
| BMI, kg/m^2^ | 25.1 (24.1 to 27.0) | 24.3 (22.0 to 26.0) | .101 |
| **Self-reported exercise** |  |  |  |
| Ex volume, min/week | 229 (193 to 265) | 213 (134 to 291) | .374 |
| **Resting metabolic rate** |  |  |  |
| MJ/day | 6.2 (5.8 to 6.5) | 5.0 (4.7 to 5.3) | < .001 |
| **Daily protein intake** |  |  |  |
| g/kg BM/day | 1.3 (1.2 to 1.5) | 1.3 (1.1 to 1.5) | .935 |
| **Biochemistry** |  |  |  |
| BGL, mmol/L | 5.1 (4.8 to5.3) | 4.7 (4.4 to 5.1) | .050 |
| Insulin, ulU/ml | 8.0 (6.6 to 9.2) | 5.6 (4.8 to 6.3) | .050 |
| IGF-1, pg/ml | 174 (27 to 377) | 108 (31 to 185) | .545 |
| Testosterone, ng/ml | 2.1 (1.6 to 2.4) | 0.2 (0.0 to 0.6) | < .001 |
| Estradiol, pg/ml | 31.3 (9.0 to 53.5) | 23.2 (1.6 to 48.2) | .657 |
| Cortisol, nmol/L | 368 (294 to 443) | 366 (473 to 360) | .658 |
| Neutrophils x10^9^ | 2.8 (2.5 to 3.1) | 2.5 (1.9 to 3.0) | .050 |
| Lymphocytes x10^9^ | 2.1 (1.8 to 2.4) | 2.1 (1.7 to 2.5) | .545 |
| Monocytes x10^9^ | 0.4 (0.3 to 0.4) | 0.3 (0.2 to 0.3) | < .001 |
| IL-2, pg/ml | 4.1 (3.2 to 5.0) | 4.4 (2.7 to 6.2) | .657 |
| IL-6, pg/ml | 5.8 (2.5 to 9.2) | 5.8 (1.2 to 10.4) | .658 |
| IL-8, pg/ml | 5.6 (3.0 to 8.3) | 6.1 (2.6 to 9.5) | .092 |
| IL-10, pg/ml | 18.0 (13.1 to 23.0) | 22.0 (14.4 to 29.1) | .140 |
| TNF-α, pg/ml | 2.3 (1.8 to 2.7) | 2.4 (1.6 to 3.2) | .828 |
| Mean (95% CI).  **Abbreviations:** BGL: blood glucose levels, BM, body mass, BMI: body mass index, IGF: insulin-like growth factor, IL: interleukin, m, metres, MJ: Megajoules, WBC: white blood cells. | | | |
